# Supplementary material for: Effects of gene dosage and development on subcortical nuclei volumes in individuals with 22q11.2 copy number variations
Source: Neuropsychopharmacology. 2024 Mar 2;49(6):1024–32. doi: 10.1038/s41386-024-01832-3 (PMC11039652; doi:10.1038/s41386-024-01832-3)
Supplement: Supplementary file 1 — Supplemental Material [file 41386_2024_1832_MOESM1_ESM.docx]

**Supplement**

**Supplemental Methods**

Participants

The total longitudinal sample consisted of 385 scans from 213 participants (5.5–49.5 years of age; n=96 22qDel baseline, 53.1% female; n=37 22qDup baseline, 45.9% female; n=80 TD controls baseline, 51.3% female), recruited from an ongoing longitudinal study at the University of California, Los Angeles (UCLA). The 22qDel and 22qDup participants all had a molecularly confirmed 22q11.2 CNV. Participants had data from between 1 and 6 time points (mean=1.81 visits, SD=1.04), separated by an average of approximately one- and three-quarter years (mean=1.76 years, SD=1.16). The three groups were statistically matched based on baseline age and sex, as well as mean number of longitudinal visits and interval between visits, using appropriate tests (ANOVA, or chi-squared). Exclusion criteria for all study participants were as follows: significant neurological or medical conditions (unrelated to 22q11.2 deletion or duplication) that might affect brain structure, history of head injury with loss of consciousness, insufficient fluency in English, and/or substance or alcohol use disorder within the past 6 months. As we aimed to include a representative cohort of CNV carriers, patients with cardiac-related and/or immune issues were not excluded, as these are common medical comorbidities in 22qDel. Healthy controls were free from significant intellectual disability and/or family history of psychotic disorder, and did not meet criteria for any psychiatric disorder, with the exception of attention deficit-hyperactivity disorder, anxiety disorders, or a past episode of depression, due to their prevalence in childhood and adolescence [1–3]. After study procedures had been fully explained, adult participants provided written consent, while participants under the age of 18 years provided written assent with the written consent of their parent or guardian. The UCLA Institutional Review Board approved all study procedures and informed consent documents.

*Clinical assessment*

At each study time point, demographic information and clinical measures were collected for each participant by trained Master's-level clinicians, supervised by a licensed clinical psychologist. Psychiatric diagnoses were established with the Structured Clinical Interview for DSM-IV (SCID), with an additional developmental disorders module [4]. Verbal IQ was assessed via the Wechsler Abbreviated Scale of Intelligence (WASI-2) Vocabulary subtest, and nonverbal IQ was assessed via the WASI-2 Matrix Reasoning subtest. Dimensional psychosis-risk and general psychiatric symptoms were assessed via the Structured Interview for Psychosis-Risk Syndromes (SIPS) [5]. For more details on study ascertainment and recruitment procedures, see Jalbrzikowski et al. 2012 and 2013 [6,7].

*Neuroimaging acquisition*

All subjects were imaged at the UCLA Center for Cognitive Neuroscience on either a Siemens TimTrio scanner (with a 12-channel head coil) or Siemens Prisma (with a 32-channel head coil). T1w scans were acquired in sagittal slices with 1mm^3^ voxels, as described in Jalbrzikowski et al. 2022 [8], using MPRAGE sequences adapted from the Alzheimer's Disease Neuroimaging Initiative (ADNI) protocol [9]. Trio and Prisma MPRAGE scans used nearly identical parameters: TR = 2.3 s, FOV = 256 mm, matrix = 240 × 256, flip angle = 9°, slice thickness = 1.20 mm, 160 slices. TE was 2.91 ms for Trio scans, and 2.94 ms for Prisma.

*Neuroimaging preprocessing*

T1w MRI scans were processed with the FreeSurfer analysis package, version 7.3.2 [10]. Scan sessions at all timepoints were first processed cross-sectionally using the recon-all anatomical segmentation pipeline [11,12]. The FreeSurfer longitudinal stream was subsequently applied, which has been shown to significantly improve reliability and statistical power in repeated-measure analyses [13]. This method generates unbiased within-subject templates using robust, inverse consistent registration, and uses these templates to improve initialization of several processing steps, such as skull-stripping, Talairach transforms, atlas registration, spherical surface maps, and parcellations [13].

*Subcortical nuclei volumes*

For each MRI scan, the FreeSurfer longitudinal segment subregions pipeline was used to estimate volumes for 25 thalamic subregions, 19 hippocampal subregions, and 9 amygdala subregions, as well as whole-structure volumes [14–17]. These methods use Bayesian inference to automatically segment T1w MRI images using probabilistic template atlases based on histological data and ultra-high-resolution *ex vivo* MRI. These segmentations have been highly validated and have been applied by multiple groups and consortia to large scale neuroimaging analyses of development and group differences in various psychiatric conditions [18–22]. For analysis, several hippocampal subregions were combined as follows: the head and tail of hippocampal CA1 were added to give a single CA1 volume, similarly for CA2/3, CA4, molecular layer, GC-ML-DG, presubiculum, and subiculum, as in Mancini et al. 2010, and Latrèche et al. 2023) [23,24]. Hippocampal regions CA2 and CA3 are combined into a single CA2/3 region in the FreeSurfer segment subregions atlas due to difficulty reliably determining the boundary between regions [14]. In the thalamus several regions were combined, as in Huang et al. 2020 [21]: the mediodorsal medial and lateral regions were combined to give one mediodorsal region; the ventral lateral anterior and posterior subregions were combined into one ventral lateral region; the ventral anterior and ventral anterior magnocellular regions were combined into one ventral anterior region; the anterior, lateral, and inferior pulvinar were combined into one pulvinar region.

The full list of analyzed regions is as follows. Amygdala: accessory basal, anterior amygdaloid, basal, central, cortical, corticoamygdaloid transition, lateral, medial, and paralaminar. Hippocampus (body + head volume summed for all relevant regions): CA1, CA2/3, CA4, granule cell and molecular layer of the dentate gyrus (GC ML DG), fimbria, hippocampal amygdala transition area, hippocampal fissure, hippocampal tail, molecular layer, parasubiculum, presubiculum, and subiculum. Thalamus: anteroventral, central lateral, central medial, centromedian, lateral geniculate, lateral posterior, laterodorsal, limitans suprageniculate, medial geniculate, medial ventral reuniens, mediodorsal (medial+lateral), parafascicular, pulvinar (anterior + lateral + inferior, excluding unreliable medial subregion), ventral anterior (ventral anterior + magnocellular), ventral lateral (posterior + anterior), ventral posterolateral, and ventromedial.

For the primary analyses, regional volumes were averaged within subjects between left and right hemispheres to reduce multiple comparisons and facilitate interpretation. See **Supplemental Results Table S4** for bilateral gene dosage analyses without this averaging step.

An example image was generated for visualization purposes (see **Figure S2**) using the FreeSurfer recon-all and segment subregions pipelines with the MNI152 template brain as the input [25].

*Quality control*

Several qualitative and quantitative approaches were taken to prevent inclusion of inaccurately estimated volumes in the analysis. First, each raw T1w image was visually assessed for quality prior to preprocessing, and excluded if quality was low (e.g., significant motion artifact, signal loss, or incomplete brain coverage). After subcortical segmentation, each image was visually checked to ensure alignment of thalamus, hippocampus, and amygdala masks with their associated structures. In four scans, bilateral thalamic volumes were excluded from further analysis because the thalamic segmentation was found to include parts of the striatum. We also excluded the medial pulvinar from further analysis in all subjects because the boundaries of the medial pulvinar mask were observed to extend beyond the thalamus in many cases. Finally, we calculated the mean and standard deviation of each subregion volume in the full cohort, and for each individual, excluded a given subregion from further analysis if the volume was greater than 3 standard deviations absolute difference from the overall group mean. Out of the total set of 33,196 regions from 386 subjects, 139 total regions across 67 subjects were flagged for exclusion by this metric. Two additional thalamic regions, the paracentral and paratenial nuclei, were excluded because the average volume was found to be less than 10 mm^3^ in each group. For the main analyses, volumes were averaged bilaterally except in the cases where a region had been excluded as an outlier in one hemisphere, in which case the non-outlier volume was used.

*Data harmonization*

To harmonize data acquired on two different scanners, we applied a longitudinal implementation of the ComBat algorithm using the longComBat package in R version 4.2.2 [26,27]. ComBat uses empirical Bayes methods to estimate and remove scanner/batch effects with increased robustness to outliers in small samples compared to general linear model approaches. ComBat was initially developed for genomics data [27], and has been subsequently adapted for neuroimaging and shown to preserve biological associations while effectively removing unwanted non-biological variation associated with site/scanner [28]. The longitudinal adaptation, which uses random effects to account for within-subject repeated measures, has been shown to further increase statistical power in longitudinal neuroimaging analyses [26]. LongComBat has been used to harmonize structural and functional MRI features in largely overlapping cohorts of individuals with 22qDel and controls [8,29]. LongComBat requires that the input data matrix not contain missing values, so for regions that were to be excluded from the final analysis, we imputed values based on the mean of that region’s volume in individuals collected on the same scanner (Trio or Prisma) with the same CNV status (22qDel, 22qDup, or TD). Excluded volumes were then set to “NA” after longComBat harmonization, prior to regression analysis.

*Gene dosage and maturational effects*

To investigate the linear effect of CNV status on subcortical volumes, and to capture the non-linear relationship between age and volume, we used a generalized additive mixed model (GAMM) approach with a linear fixed effect for gene dosage, which was numerically coded based on CNV status: 22qDel=1, TD=2, and 22qDup=3 copies of the 22q11.2 locus. Age was modeled with separate flexible thin plate regression splines in each group [8], restricted to exactly 2 degrees of freedom (DoF) per group to facilitate comparison [30]. GAMMs are a nonlinear extension of mixed effects regression, allowing for repeat visits from the same participant to be modeled with a random intercept [31]. Biological sex and site were also included as fixed effects in each model. Total intracranial volume (ICV) was included as a fixed effect in all models except where ICV was the dependent variable. Models were fit with restricted estimation of maximum likelihood, using mgcv in R [32]. Prior to testing, the ComBat-adjusted volume for each region was normalized based on the TD group mean and standard deviation.

An example R mgcv::gam formula for a GAMM to model the volume in a given region is:

*volume ~ s(age, by = group, bs = "tp", k = 3, fx = TRUE) + gene_dosage + sex + icv + site +  s(subject_id, bs = "re", k = 3)*

In which regional volume is predicted by an age smooth in each group using thin plate regression splines with exactly k-1=2 DoF per smooth, with linear fixed effects for gene dosage, sex, total ICV, site, and a random effect for subject ID accounting for repeat visits.

Gene dosage effects were tested using this model for total ICV and whole thalamus, hippocampus, and amygdala volumes, followed by each of the 38 subregions. All tests were corrected for multiple comparisons using the standard False Discovery Rate (FDR) at a threshold of *q*<0.05 across the 42 volumes [33].

To characterize maturational trajectories, p-values for the non-linear effect of age in each group were computed and evaluated at *q*<0.05 across all 126 models. Age ranges of significant difference between CNV groups and controls were computed from the 95% confidence interval for the difference in curves.

*Secondary analyses*

Several secondary analyses were performed to complement the primary gene dosage analyses. Regional volume differences compared to the TD group were tested separately for 22qDel and 22qDup groups. Gene dosage analyses were repeated with antipsychotic medication status as an additional covariate. Additional models of antipsychotic status effects on volume were tested in only 22qDel, which was the only group with multiple more than 10% of participants taking antipsychotic medication. Gene dosage analyses were also repeated without averaging structures bilaterally to detect any asymmetric hemispheric effects. Secondary analyses of the interaction between sex and gene dosage on brain volumes were also tested.

*Cognition and symptom analyses*

Motivated by existing literature relating low hippocampal tail volume to verbal learning impairment in 22qDel [23], we assessed verbal and non-verbal IQ (Wechsler Abbreviated Scale of Intelligence (WASI-2) Vocabulary and Matrix subtest scaled scores) for associations with hippocampal tail volume in each group. A recent study of hippocampal volumes in 22qDel from another research group found that decreased hippocampal tail volume was associated with impaired development of verbal learning [23]. Here we sought to replicate that finding and extend to 22qDup. In each group (22qDel, 22qDup, and TD) we tested a linear mixed model predicting verbal and non-verbal IQ (WASI-2 Vocabulary Verbal and Matrix Reasoning subtest scaled scores) from hippocampal tail volume, controlling for sex and scanner, with a random intercept for subject ID. See **Supplemental Figure S3** for results.

Because 22qDel is associated with psychosis risk, we tested relationships between psychosis risk symptoms and subcortical volumes in the 22qDel group. Specifically, we tested models relating positive symptom scores from the Structured Interview for Psychosis-Risk Syndromes (SIPS) [5] to volume across each region, controlling for age, age^2^, sex, and scanner, with a random intercept for each individual participant. We also similarly tested models relating volume to categorical diagnosis of Psychosis Risk Symptoms, operationalized here as having any score of 3 or greater (i.e., prodromal range) on any SIPS positive symptom item.

**Supplemental Results**


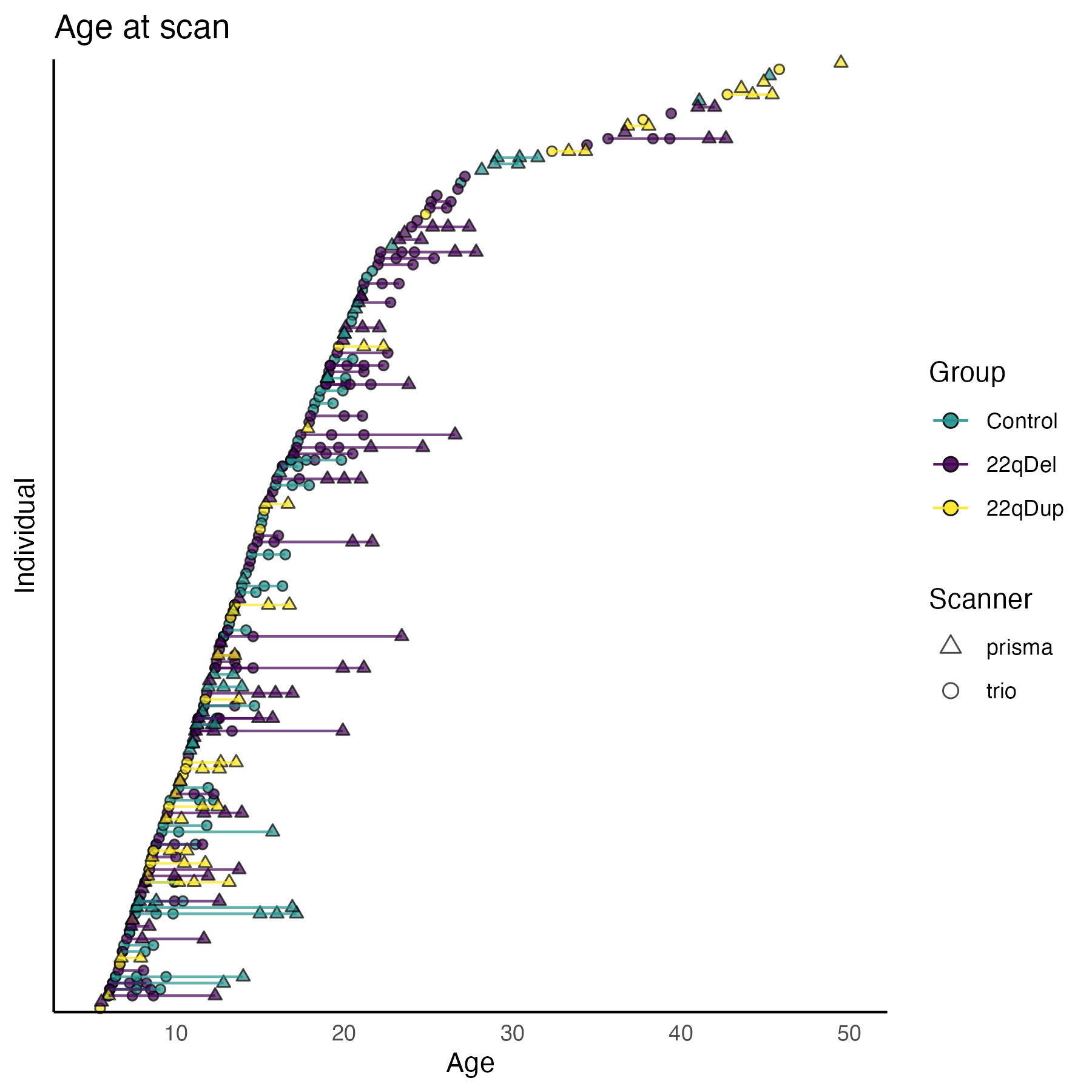


**Figure S1. Participant age distribution.** Typically developing controls in green, 22qDel in purple, and 22qDup in yellow, with lines connecting follow-up visits from the same individual. Scanner type (Siemens Trio or Prisma) indicated by circle or triangle, respectively.


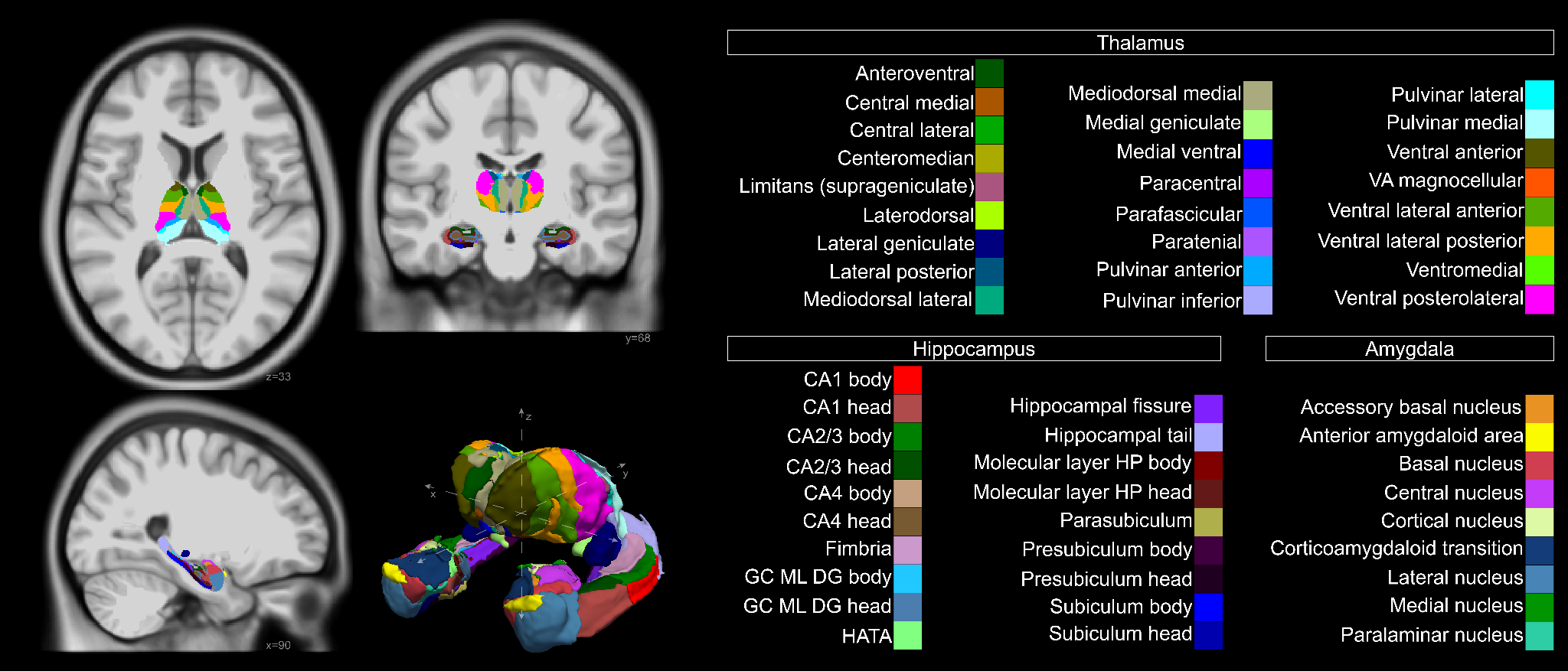


**Figure S2**. **Anatomical parcellation of thalamus, hippocampus, and amygdala nuclei.** *Left:* axial, coronal, and sagittal slices, and 3D reconstruction of structures. Generated for visualization purposes using the FreeSurfer recon-all and segment subregions pipelines with the MNI152 template brain as the input. *Right:* structure names and color key. Abbreviations: VA = Ventral Anterior, CA = Cornu Ammonis (areas 1, 3 and 4), GC ML DG = Granule Cell and Molecular Layer of the Dentate Gyrus, HATA = Hippocampus Amygdala Transition Area, HP = Hippocampus.

**
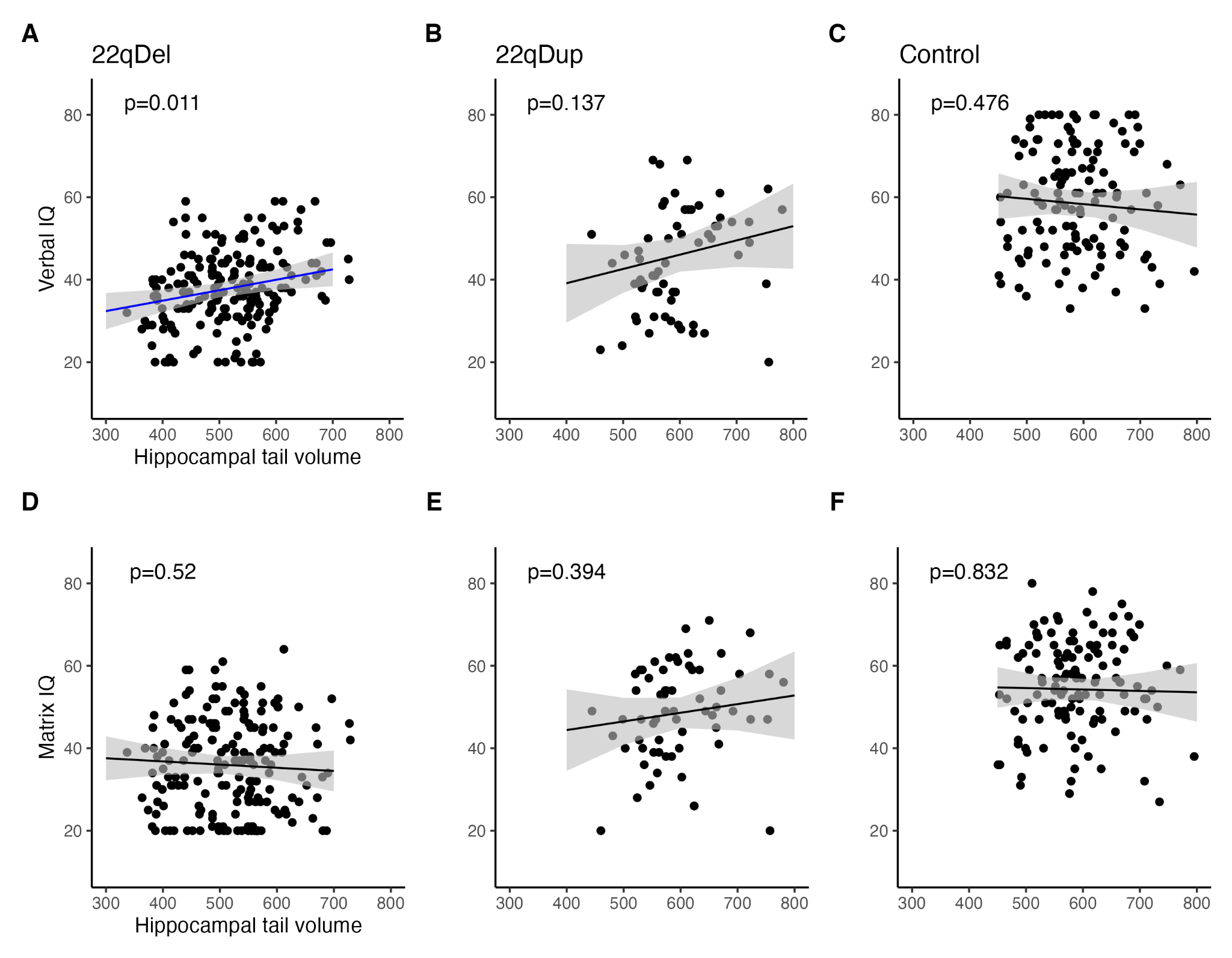
**

**Figure S3. Relationships between IQ subtests and hippocampal tail volume. A-C**) WASI-2 Verbal IQ scaled scores were significantly predicted by hippocampal tail volume controlling for sex, site, and participant in 22qDel (*beta*=1.86, *p*=0.011) but not 22qDup or TD controls. **D-F**) Matrix Reasoning (Nonverbal IQ) subscale scores were not related to hippocampal tail volume in any group. Adding Nonverbal IQ as a covariate in the model predicting Verbal IQ from hippocampal tail volumes increased the strength of the verbal IQ relationship in 22qDel (*beta*=2.07, *p*=0.0018).


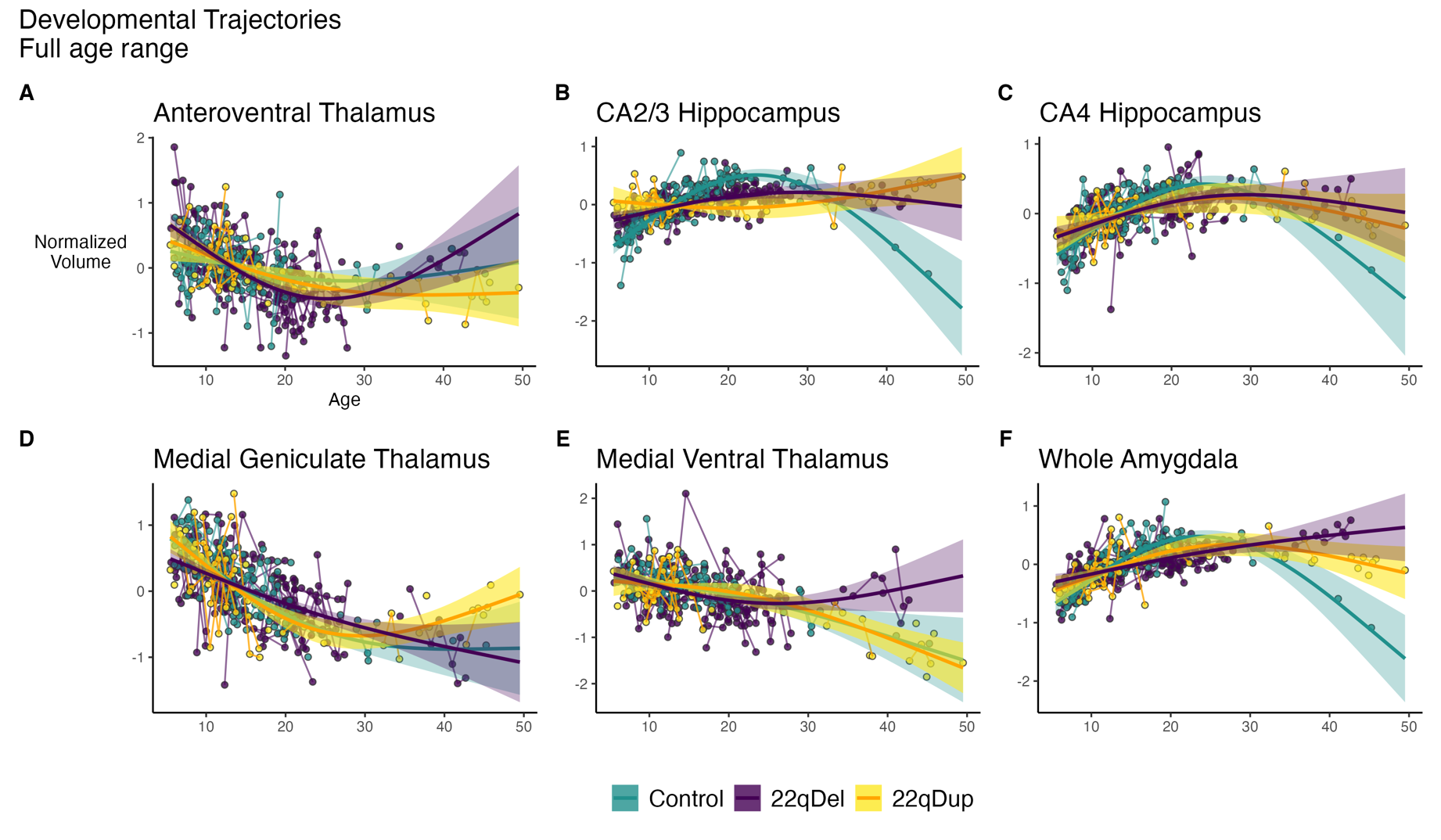


**Figure S4. Age curves with partial residuals.** The same curves as main text **Figure 3**, with the addition of scatter plots for partial residuals for each scan, with repeat scans from the same individual connected with lines.


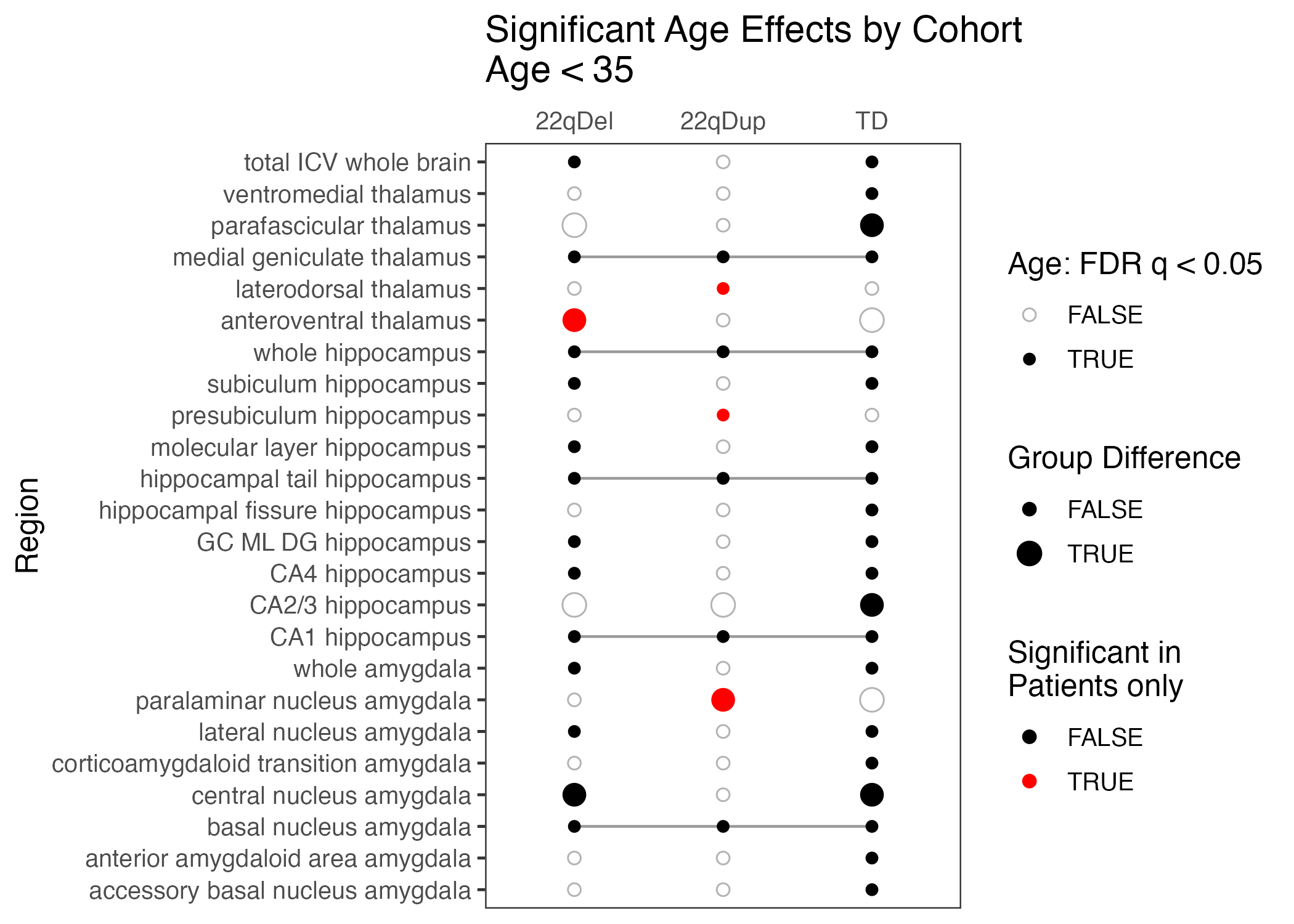


**Figure S5. Summary of age effects on subcortical volumes for participants under 35 years of age.** Same as main text Figure 2, for models with the maximum age restricted to 35 years to exclude the relatively small set of subjects between ages 35 and 49.5 (n=13 excluded across all groups). Key results are similar: all three groups show significant age effects in the medial geniculate thalamus, 22qDel show steeper declines in anteroventral thalamus, and both 22qDel and 22qDup show flattened hippocampal CA2/3 development.

**
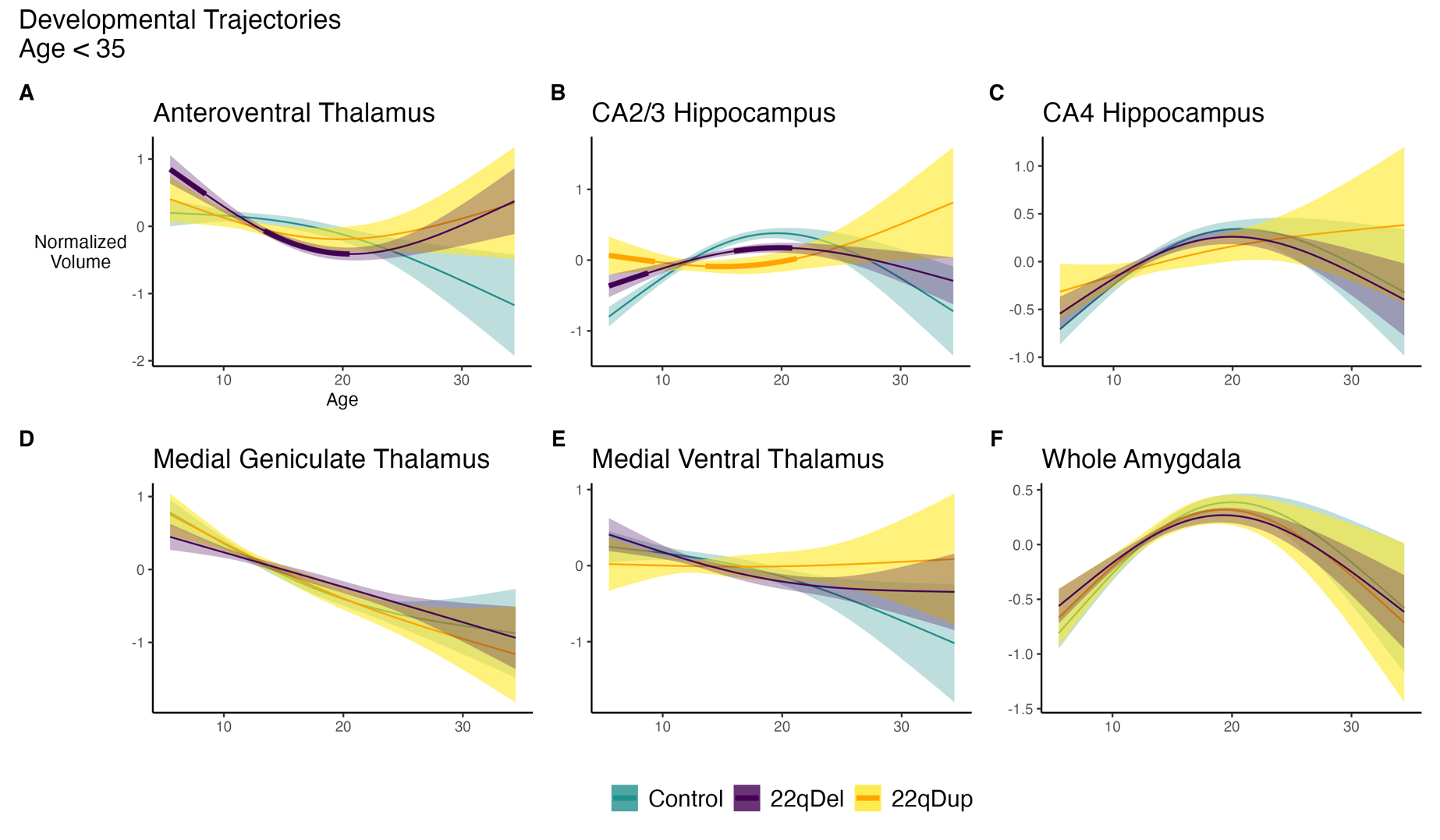
**

**Figure S6. Age curves with group differences, under 35 years old.** The same plots as main text **Figure 3**, restricted to participants under 35 years of age. Key results are similar: all three groups show significant age effects in the medial geniculate thalamus, 22qDel show steeper declines in anteroventral thalamus, and both 22qDel and 22qDup show flattened hippocampal CA2/3 development.

**
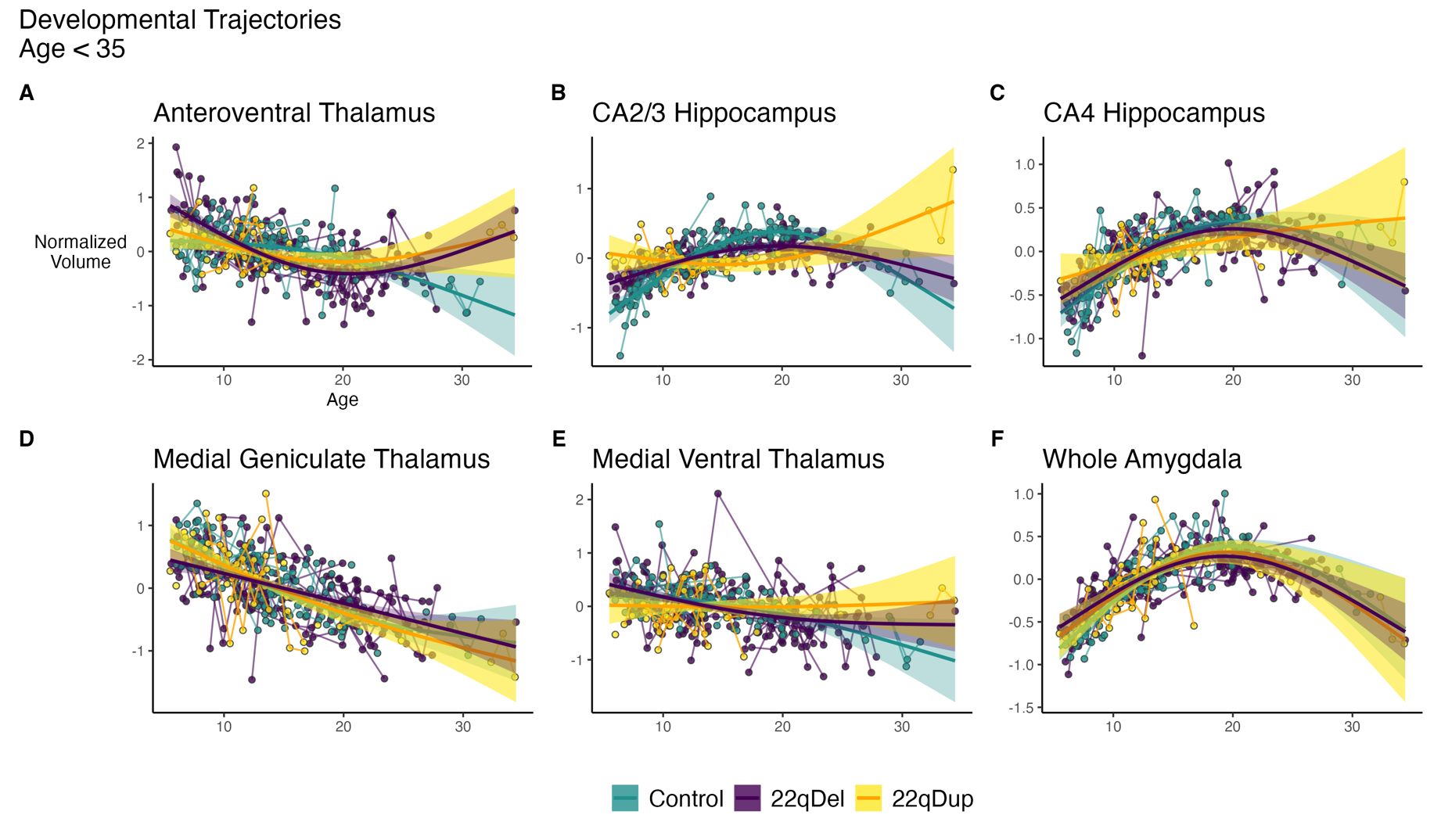
**

**Figure S7. Age curves with partial residuals, under 35 years old.** The same plots as supplemental **Figure S4**, restricted to participants under 35 years of age.


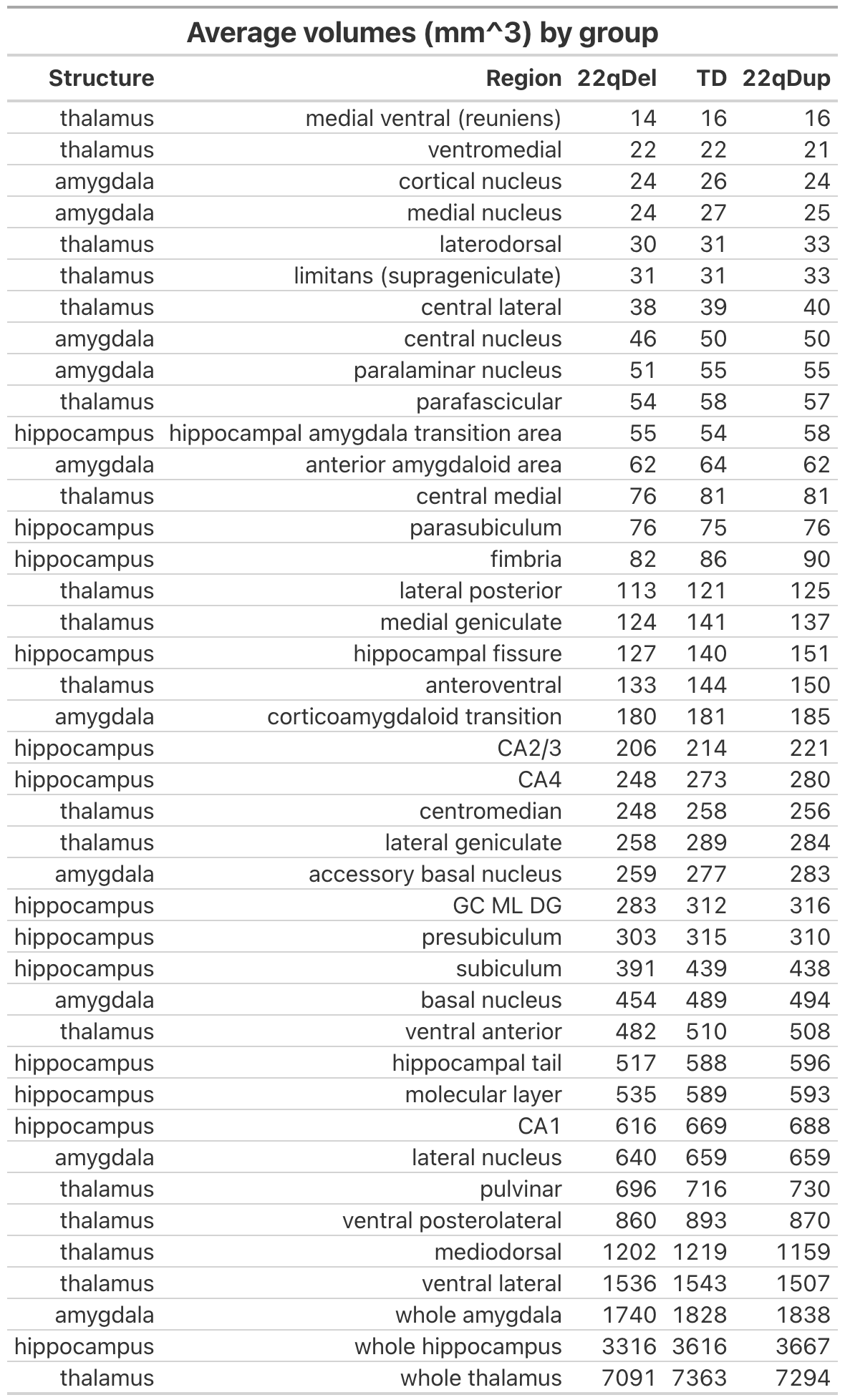


**Table S1. Average volumes by group.** Reported for whole thalamus, hippocampus, and amygdala and all analyzed subregions. Sorted by ascending volume in the 22qDel group.

**
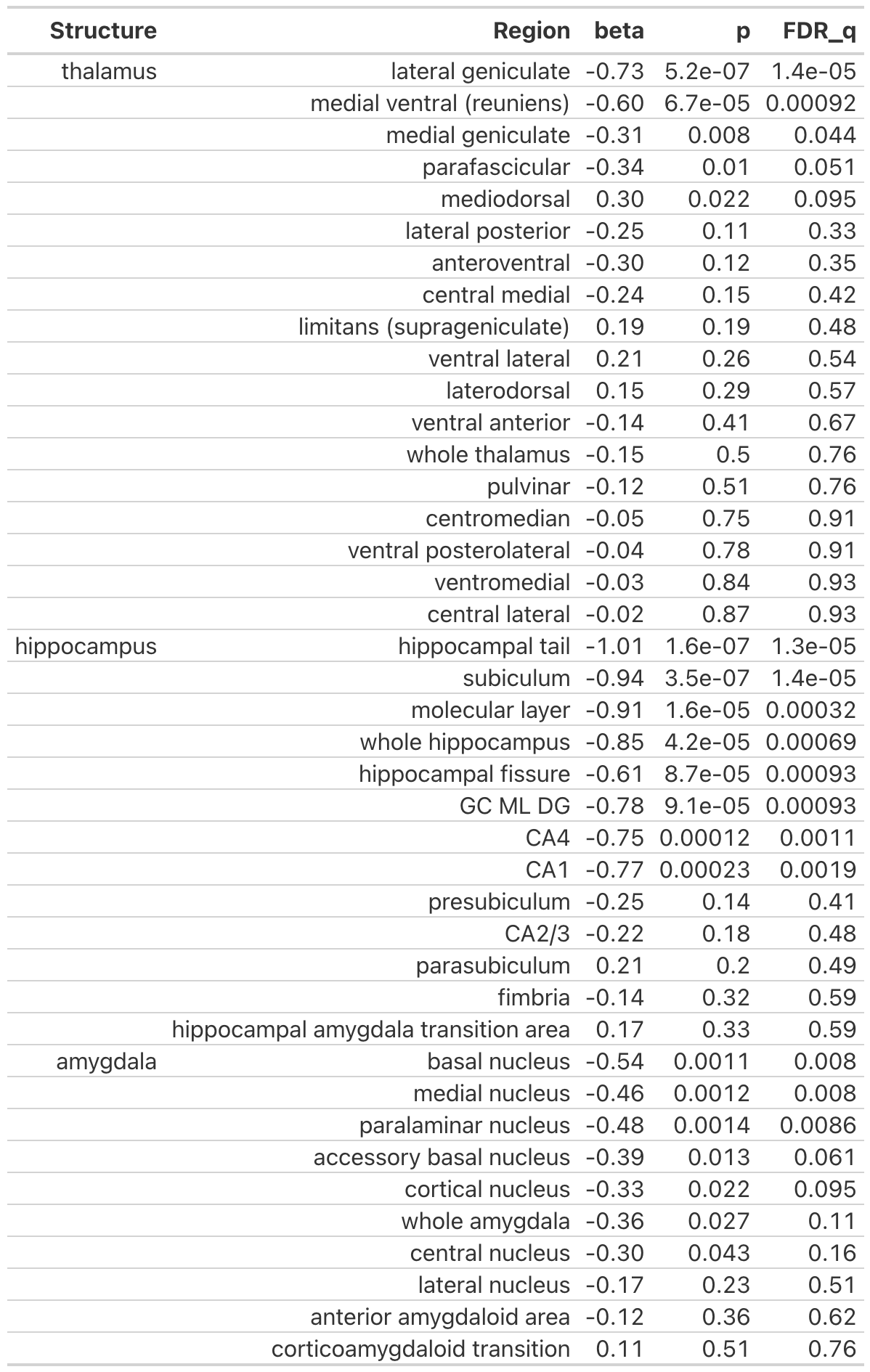
**

**Table S2. 22qDel versus TD comparisons.** General additive mixed models (GAMMs) linearly predicting normalized brain volumes from group (22qDel or TD), controlling for sex, site, participant, total brain volume, and non-linear age in the full longitudinal sample.


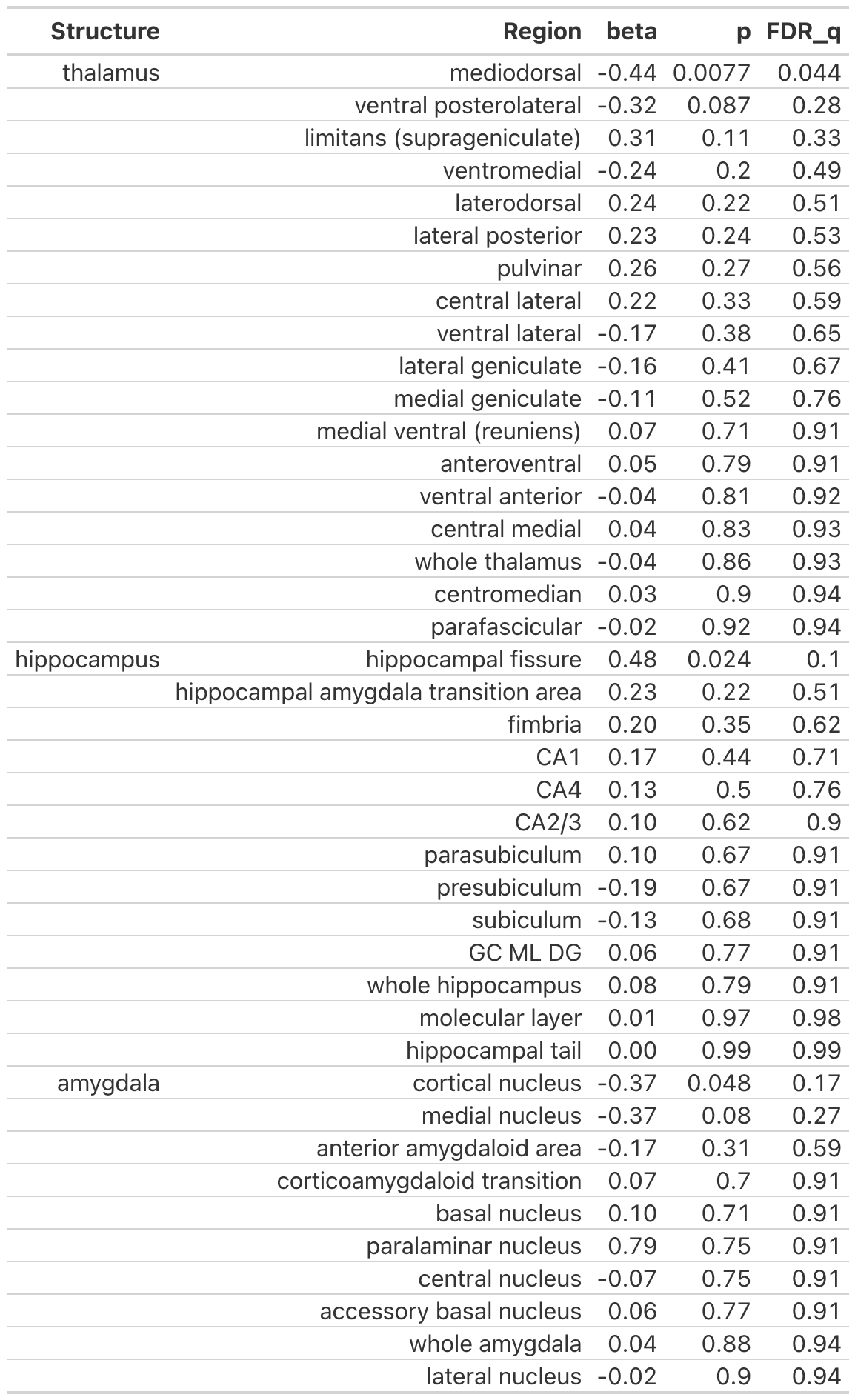

**Table S3. 22qDup versus TD comparisons.** General additive mixed models (GAMMs) linearly predicting normalized brain volumes from group (22qDup or TD), controlling for sex, site, participant, total brain volume, and non-linear age in the full longitudinal sample.


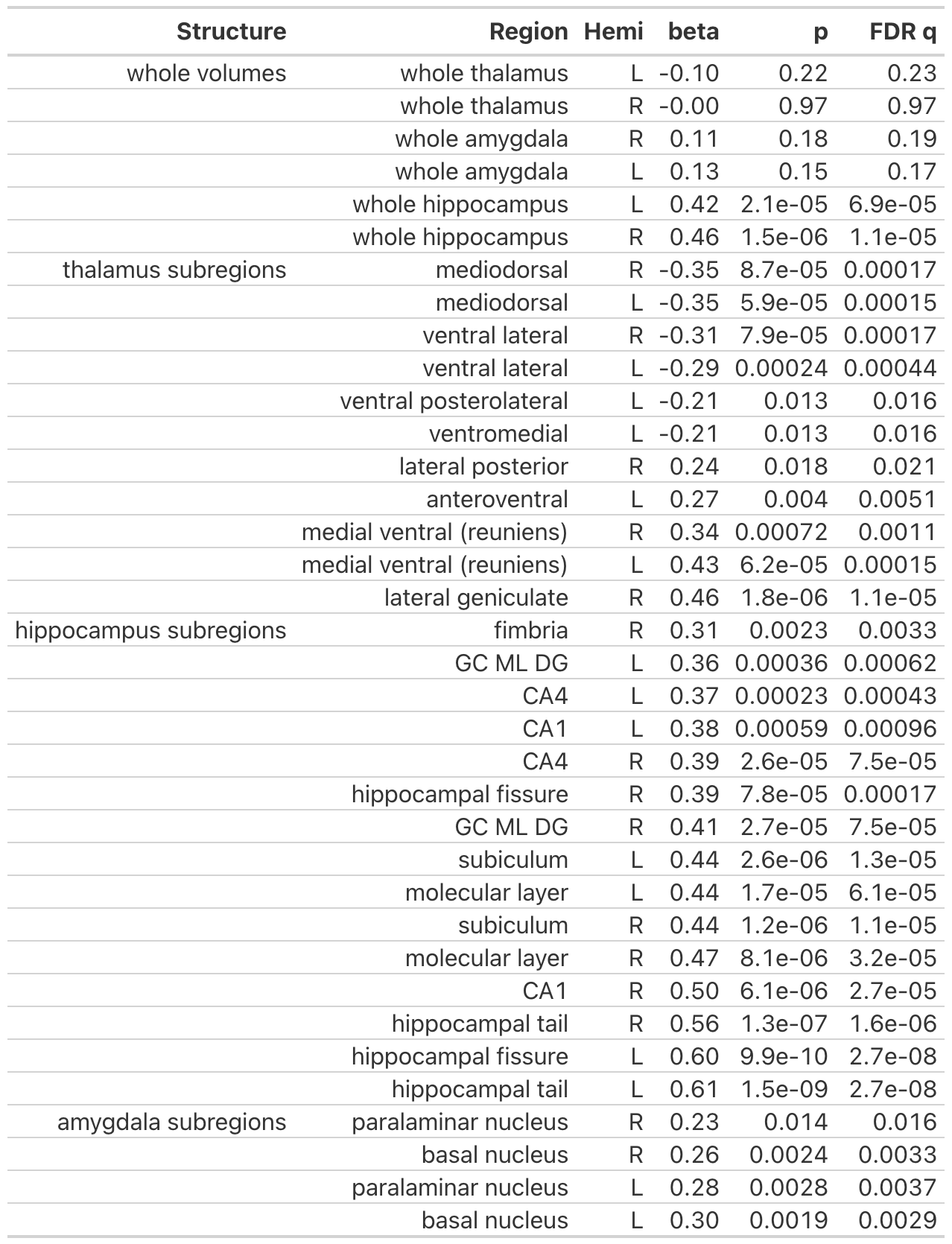


**Table S4. Gene dosage effects in individual hemispheres.** Repeat of main analyses without averaging regions bilaterally, showing highly similar effects of gene dosage on regional volume in the left and right hemispheres. Whole structures and subregions with FDR q < 0.05 are listed in this table.

**
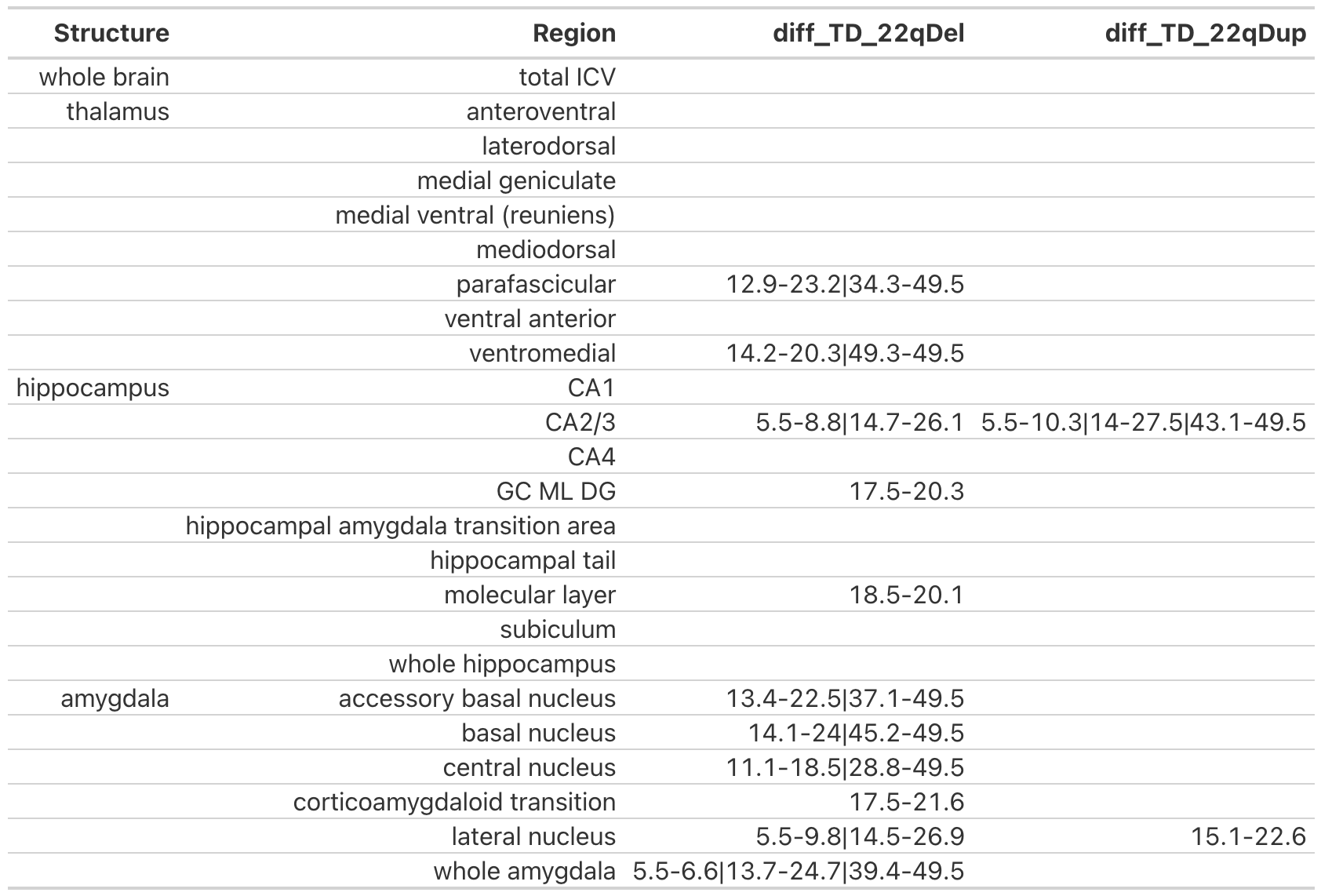
**

**Table S5. Age ranges with significant differences between CNV carriers and controls.** Age periods with group difference based on 95% confidence interval (CI), multiple discontinuous ranges separated with “|”. diff_TD_22qDel lists differences between 22qDel and TD curves, diff_TD_22qDup shows the same for 22qDup.

**
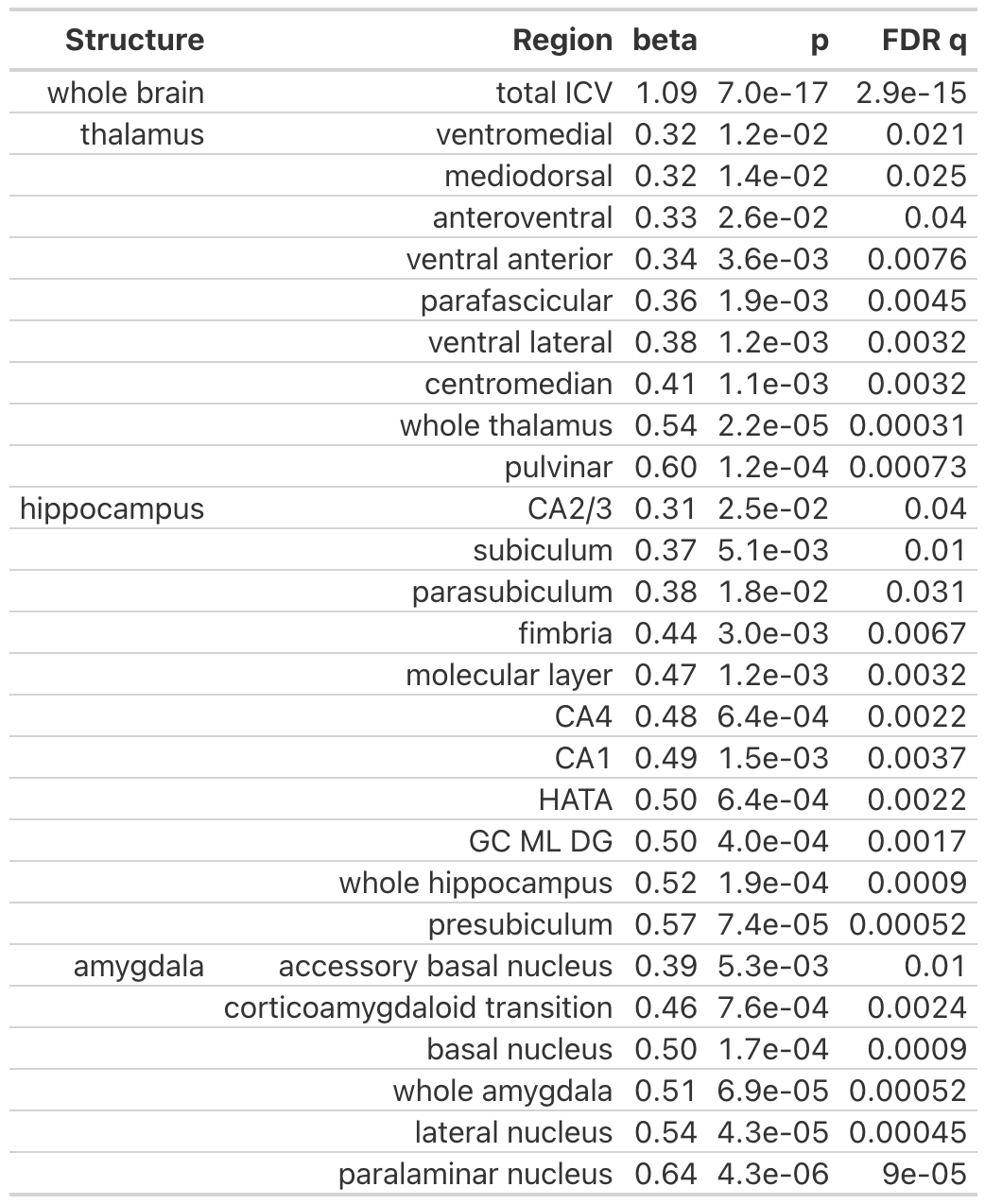
**

**Table S6. Main effects of sex**. Regions with an FDR significant main effect of sex in the GAMM used for the primary gene dosage analysis.

**
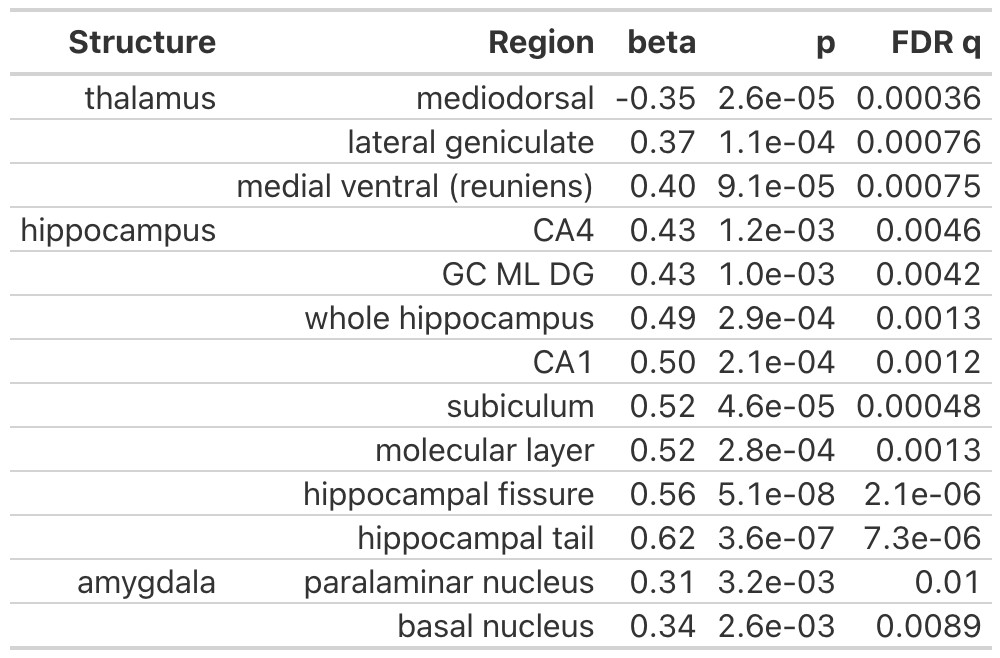
**

**Table S7. Gene dosage effects controlling for antipsychotic medication.** Repeat of gene dosage volume analysis with the addition of a covariate coding whether or not each participant was taking antipsychotic medication at the time of the scan. Regions with FDR q < 0.05 are listed in this table.

**Supplemental References**

1. Ghandour RM, Sherman LJ, Vladutiu CJ, Ali MM, Lynch SE, Bitsko RH, et al. Prevalence and Treatment of Depression, Anxiety, and Conduct Problems in US Children. J Pediatr. 2019;206:256-267.e3.

2. Sayal K, Prasad V, Daley D, Ford T, Coghill D. ADHD in children and young people: prevalence, care pathways, and service provision. Lancet Psychiatry. 2018;5:175–186.

3. Thapar A, Collishaw S, Pine DS, Thapar AK. Depression in adolescence. The Lancet. 2012;379:1056–1067.

4. First MB, Gibbon M. The Structured Clinical Interview for DSM-IV Axis I Disorders (SCID-I) and the Structured Clinical Interview for DSM-IV Axis II Disorders (SCID-II). Compr. Handb. Psychol. Assess. Vol 2 Personal. Assess., Hoboken, NJ, US: John Wiley & Sons, Inc.; 2004. p. 134–143.

5. Miller TJ, McGlashan TH, Rosen JL, Somjee L, Markovich PJ, Stein K, et al. Prospective diagnosis of the initial prodrome for schizophrenia based on the Structured Interview for Prodromal Syndromes: preliminary evidence of interrater reliability and predictive validity. Am J Psychiatry. 2002;159:863–865.

6. Jalbrzikowski M, Carter C, Senturk D, Chow C, Hopkins JM, Green MF, et al. Social Cognition in 22q11.2 Microdeletion Syndrome: Relevance to Psychosis. Schizophr Res. 2012;142:99–107.

7. Jalbrzikowski M, Jonas R, Senturk D, Patel A, Chow C, Green MF, et al. Structural abnormalities in cortical volume, thickness, and surface area in 22q11.2 microdeletion syndrome: Relationship with psychotic symptoms. NeuroImage Clin. 2013;3:405–415.

8. Jalbrzikowski M, Lin A, Vajdi A, Grigoryan V, Kushan L, Ching CRK, et al. Longitudinal trajectories of cortical development in 22q11.2 copy number variants and typically developing controls. Mol Psychiatry. 2022:1–10.

9. Jack Jr. CR, Bernstein MA, Fox NC, Thompson P, Alexander G, Harvey D, et al. The Alzheimer’s disease neuroimaging initiative (ADNI): MRI methods. J Magn Reson Imaging. 2008;27:685–691.

10. FreeSurferMethodsCitation. https://surfer.nmr.mgh.harvard.edu/fswiki/FreeSurferMethodsCitation. Accessed 27 February 2023.

11. Fischl B, Salat DH, Busa E, Albert M, Dieterich M, Haselgrove C, et al. Whole brain segmentation: automated labeling of neuroanatomical structures in the human brain. Neuron. 2002;33:341–355.

12. Fischl B, van der Kouwe A, Destrieux C, Halgren E, Ségonne F, Salat DH, et al. Automatically parcellating the human cerebral cortex. Cereb Cortex N Y N 1991. 2004;14:11–22.

13. Reuter M, Schmansky NJ, Rosas HD, Fischl B. Within-subject template estimation for unbiased longitudinal image analysis. Neuroimage. 2012;61:1402–1418.

14. Iglesias JE, Augustinack JC, Nguyen K, Player CM, Player A, Wright M, et al. A computational atlas of the hippocampal formation using ex vivo, ultra-high resolution MRI: Application to adaptive segmentation of in vivo MRI. NeuroImage. 2015;115:117–137.

15. Iglesias JE, Van Leemput K, Augustinack J, Insausti R, Fischl B, Reuter M, et al. Bayesian longitudinal segmentation of hippocampal substructures in brain MRI using subject-specific atlases. NeuroImage. 2016;141:542–555.

16. Iglesias JE, Insausti R, Lerma-Usabiaga G, Bocchetta M, Van Leemput K, Greve DN, et al. A probabilistic atlas of the human thalamic nuclei combining ex vivo MRI and histology. Neuroimage. 2018;183:314–326.

17. Saygin ZM, Kliemann D, Iglesias JE, van der Kouwe AJW, Boyd E, Reuter M, et al. High-resolution magnetic resonance imaging reveals nuclei of the human amygdala: manual segmentation to automatic atlas. NeuroImage. 2017;155:370–382.

18. Sämann PG, Iglesias JE, Gutman B, Grotegerd D, Leenings R, Flint C, et al. FreeSurfer-based segmentation of hippocampal subfields: A review of methods and applications, with a novel quality control procedure for ENIGMA studies and other collaborative efforts. Hum Brain Mapp. 2022;43:207–233.

19. Grace S, Rossetti MG, Allen N, Batalla A, Bellani M, Brambilla P, et al. Sex differences in the neuroanatomy of alcohol dependence: hippocampus and amygdala subregions in a sample of 966 people from the ENIGMA Addiction Working Group. Transl Psychiatry. 2021;11:156.

20. Weeland CJ, Kasprzak S, de Joode NT, Abe Y, Alonso P, Ameis SH, et al. The thalamus and its subnuclei—a gateway to obsessive-compulsive disorder. Transl Psychiatry. 2022;12:70.

21. Huang AS, Rogers BP, Sheffield JM, Jalbrzikowski ME, Anticevic A, Blackford JU, et al. Thalamic Nuclei Volumes in Psychotic Disorders and in Youths With Psychosis Spectrum Symptoms. Am J Psychiatry. 2020;177:1159–1167.

22. Hoang D, Lizano P, Lutz O, Zeng V, Raymond N, Miewald J, et al. Thalamic, Amygdalar, and Hippocampal Nuclei Morphology and their Trajectories in First Episode Psychosis: A Preliminary Longitudinal Study. Psychiatry Res Neuroimaging. 2021;309:111249.

23. Latrèche C, Maeder J, Mancini V, Bortolin K, Schneider M, Eliez S. Altered developmental trajectories of verbal learning skills in 22q11.2DS: associations with hippocampal development and psychosis. Psychol Med. 2022:1–10.

24. Mancini V, Sandini C, Padula MC, Zöller D, Schneider M, Schaer M, et al. Positive psychotic symptoms are associated with divergent developmental trajectories of hippocampal volume during late adolescence in patients with 22q11DS. Mol Psychiatry. 2020;25:2844–2859.

25. Fonov V, Evans AC, Botteron K, Almli CR, McKinstry RC, Collins DL. Unbiased average age-appropriate atlases for pediatric studies. NeuroImage. 2011;54:313–327.

26. Beer JC, Tustison NJ, Cook PA, Davatzikos C, Sheline YI, Shinohara RT, et al. Longitudinal ComBat: A method for harmonizing longitudinal multi-scanner imaging data. NeuroImage. 2020;220:117129.

27. Johnson WE, Li C, Rabinovic A. Adjusting batch effects in microarray expression data using empirical Bayes methods. Biostatistics. 2007;8:118–127.

28. Fortin J-P, Cullen N, Sheline YI, Taylor WD, Aselcioglu I, Cook PA, et al. Harmonization of cortical thickness measurements across scanners and sites. NeuroImage. 2018;167:104–120.

29. Schleifer CH, O’Hora KP, Jalbrzikowski M, Bondy E, Kushan-Wells L, Lin A, et al. Longitudinal development of thalamocortical functional connectivity in 22q11.2 deletion syndrome. 2023:2023.06.22.546178.

30. Larsen B, Bourque J, Moore TM, Adebimpe A, Calkins ME, Elliott MA, et al. Longitudinal Development of Brain Iron Is Linked to Cognition in Youth. J Neurosci. 2020;40:1810–1818.

31. Hastie TJ. Generalized Additive Models. Stat. Models S, Routledge; 1992.

32. Wood SN. Generalized Additive Models: An Introduction with R, Second Edition. 2nd ed. Boca Raton: Chapman and Hall/CRC; 2017.

33. Benjamini Y, Hochberg Y. Controlling the False Discovery Rate: A Practical and Powerful Approach to Multiple Testing. J R Stat Soc Ser B Methodol. 1995;57:289–300.
